# Genome-Scale Reconstruction of the Human Astrocyte Metabolic Network

**DOI:** 10.3389/fnagi.2017.00023

**Published:** 2017-02-13

**Authors:** Cynthia A. Martín-Jiménez, Diego Salazar-Barreto, George E. Barreto, Janneth González

**Affiliations:** ^1^Departamento de Nutrición y Bioquímica, Facultad de Ciencias, Pontificia Universidad JaverianaBogotá, Colombia; ^2^Instituto de Ciencias Biomédicas, Universidad Autónoma de ChileSantiago, Chile

**Keywords:** astrocyte, genomic-scale metabolic network, model, systems biology, ischemia

## Abstract

Astrocytes are the most abundant cells of the central nervous system; they have a predominant role in maintaining brain metabolism. In this sense, abnormal metabolic states have been found in different neuropathological diseases. Determination of metabolic states of astrocytes is difficult to model using current experimental approaches given the high number of reactions and metabolites present. Thus, genome-scale metabolic networks derived from transcriptomic data can be used as a framework to elucidate how astrocytes modulate human brain metabolic states during normal conditions and in neurodegenerative diseases. We performed a Genome-Scale Reconstruction of the Human Astrocyte Metabolic Network with the purpose of elucidating a significant portion of the metabolic map of the astrocyte. This is the first global high-quality, manually curated metabolic reconstruction network of a human astrocyte. It includes 5,007 metabolites and 5,659 reactions distributed among 8 cell compartments, (extracellular, cytoplasm, mitochondria, endoplasmic reticle, Golgi apparatus, lysosome, peroxisome and nucleus). Using the reconstructed network, the metabolic capabilities of human astrocytes were calculated and compared both in normal and ischemic conditions. We identified reactions activated in these two states, which can be useful for understanding the astrocytic pathways that are affected during brain disease. Additionally, we also showed that the obtained flux distributions in the model, are in accordance with literature-based findings. Up to date, this is the most complete representation of the human astrocyte in terms of inclusion of genes, proteins, reactions and metabolic pathways, being a useful guide for *in-silico* analysis of several metabolic behaviors of the astrocyte during normal and pathologic states.

## Introduction

Astrocytes are the most numerous glial cells found in the vertebrate brain (Verkhratsky et al., 2014). Essentially, these cells have a major role in the functions of the central nervous system and participate in key processes such as neurogenesis (Figueiredo et al., 2014), synaptogenesis (Hughes et al., 2010), neuro-inflammation (van Dijk et al., 2015) and neuro-modulation. Considering the strong metabolic cooperation that exists between neurons and astrocytes (Barreto et al., 2011a), increasing evidence has shown the importance of astrocytic dysfunction in the pathophysiology of neurological disorders, such as epilepsy, stroke, inflammatory diseases, Alzheimer's disease, Parkinson's disease, and amyotrophic lateral sclerosis (Cabezas et al., 2012; Guillamón-Vivancos et al., 2015). The role played by astrocytes in brain functions has not been extensively assessed, despite evidence suggesting that astrocyte dysfunction might play a unique role in promoting multiple brain disorders (Barreto et al., 2011b; Cabezas et al., 2012; Acaz-Fonseca et al., 2014). Thus, the manner in which astrocytes respond to physiological or pathological conditions are likely important in determining the outcome of a brain damage. Hence, the metabolic interactions in astrocytes require more investigation for a better understanding of the metabolic activity of these cells in response to different conditions that involved the brain. The identification of metabolic pathways of astrocytes is a difficult task that can be simplified by mathematical models. Mathematical models allow the systematic analysis of complex biological phenomena. System approaches integrates mathematical model formulations with computational modellings, that could represent the biochemical knowledge used to strengthen experimental results (Palsson, 2009; Hyduke et al., 2013). The expression of biological phenomena using mathematical models has been used to understand how information, energy and matter behaves in the cell and to get an efficient statistical inference in experimental research by studying the dynamical behavior of cell (Najafi et al., 2014; Vanlier et al., 2014; Özcan and Çakır, 2016; Rajkumar et al., 2016).

A metabolic reconstruction is built from a variety of biological knowledge source such as integration of high-throughput omic data, biological data bases, literature (Najafi et al., 2014). The goal is to obtain a robust model, which can be used to infer new properties; for example, the genome-scale metabolic human known as Recon and published in 2015 has been one of the most annotated model for human metabolism (Thiele et al., 2013), and it has been used in different areas including synthetic biology (He et al., 2016), metabolomics and cancer research (Petoukhov et al., 2014).

Moreover, the genome-scale network reconstructions have been used in deciphering the scope and functional implications of the regulated and dysregulated metabolism in different systems, making themselves an useful and promising tool to determine the role of cells in metabolic responses during different stimuli and insults (Weckwerth and Morgenthal, 2005). Some examples of the use of gene microarrays to model genome-scale metabolic reconstructions have been recently reported (Palsson, 2009; Hyduke et al., 2013; Aung et al., 2014). In this regard, it is possible to analyze and predict metabolic behaviors at different cell levels in order to determine how an individual component interacts in the systems and influences on the total cell function (Schlage et al., 2011; Agren et al., 2012; Sertbaş et al., 2014).

Specific metabolic models applied to different human brain systems have been developed previously, including astrocyte-neuron coupled models in an environment that simulates metabolic variations experienced in different neurodegenerative diseases (Cakir et al., 2007; Lewis et al., 2010; Calvetti and Somersalo, 2012, 2013). In this aspect, astrocyte models have been developed to determine calcium waves during astrocyte interaction with other cells (Gibson et al., 2008). Additionally, Cakir et al. reconstructed a brain metabolic network based on a previous model by adding transcriptional data of six neurodegenerative diseases. This model included 630 metabolic reactions, of which 253 of the reactions took place in neurons while 299 of them were astrocytic. The model predicted transcriptional factors such as USF1, SP1, and FOX that have regulatory roles in the neurodegenerative diseases. To date, most of reconstructed metabolic networks strive to include reactions involved in astrocytes by literature revision. Therefore, many genes and metabolic reactions are not included in these models. Since these models contain small and specific metabolic systems, the main goal of our study was to generate the most complete genome-scale metabolic reconstruction (GSMM) of a human astrocyte based on genomic, transcriptomic, biochemical and physiological data.

The work flow described in our study integrates brain-specific omics data and literature-based manual curation to generate high quality GSMM of human astrocyte. The model includes 5,659 reactions and 5,007 metabolites which are distributed in eight cell compartments (Extracellular, cytoplasm, mitochondria, endoplasmic reticle, Golgi apparatus, lysosome, peroxisome and nucleus). The reconstruction covers a large number of metabolic reactions that belong in most part to the energetic metabolism including carbohydrate metabolism, amino acid metabolism, xenobiotics biodegradation, nucleotide metabolism and metabolism of cofactors and vitamins. Furthermore, the model was validated using a flux Balance Analysis (FBA), for prediction of fluxes in the simulation. Additionally, network evaluation was performed to examine if the model was capable to generate the common astrocytic metabolites reported by the literature (Zwingmann et al., 2000; Shanker et al., 2001; Lebon et al., 2002).

On the other hand, mathematical modeling approximations under restrictions can help to understand the metabolic changes that occur in different pathologies (Lewis et al., 2010; Zhao and Huang, 2011; Bordbar and Palsson, 2012). Such models have been a helpful tool for studying roles of different cells in pathological states. In these aspect, we simulated and determined the predictive accuracy of our model during ischemic disease, through a process called random sampling, which allowed to determine the range of possible steady-state fluxes in the network. Ischemic related pathologies are one of the leading causes of long term impairment and death (Arauz and Ruíz-Franco, 2012). During ischemic brain damage, the astrocytes have diverse and important functions that involve metabolic responses important for preventing the extent of brain damage. The fluxes in ischemic astrocyte network were coherent with experimental results reported in literature. Therefore, this metabolic model serve as an effective predictive model that represents a large- scale hypothesis of how astrocyte work in different states.

To our knowledge, this is the first model of astrocytic metabolism at genomic scale that could be used for future studies on the integration of multiple biological data, identification of new therapeutic targets, recognition of biomarkers and development of new and improved neuroprotective strategies for common diseases that involve nervous system. Additionally, this reconstruction is fully compatible with Human metabolic atlas, which will facilitate studies of astrocyte-cells interactions in the context of neurodegenerative diseases.

## Materials and methods

**Figure 1:**
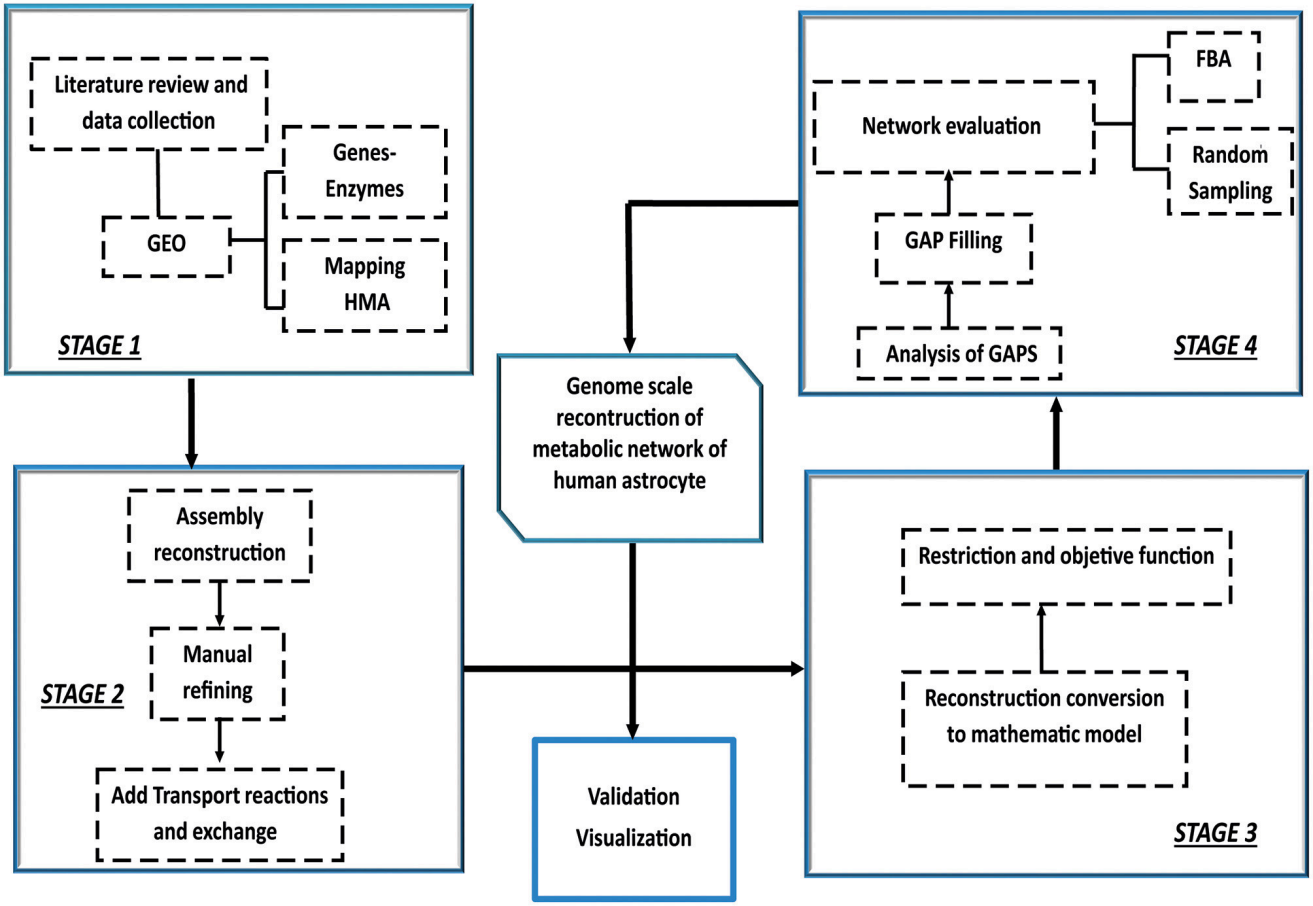
**Iterative procedure for reconstruction of the genome scale metabolic network of human astrocyte**. Construction, validation and analysis of the Genomic-Scale Metabolic Model of Astrocyte, was developed in four stages following the methodology proposed by Thiele and Palsson (2010). The omics data set was integrated with human metabolic network (HMA). Following the initial reconstruction process, the reconstruction is converted into a mathematical model format that can be used for computation. Afterwards, flux balance analysis (FBA) and random sampling was used to adjust and evaluate restrictions imposed on the model under different conditions. Finally, the analysis predictions were compared and validated against experimental studies.

Construction, validation and analysis of the Genomic-Scale Metabolic Model of Astrocyte was developed in four stages following the methodology proposed by Thiele and Palsson (2010) Manuscript (2011) (Figure 1).

### Stage 1: preliminary reconstruction of the genome scale metabolic network model

On this first stage, astrocyte genomic, transcriptomic, biochemical and physiological data information was collected according to Baart y Martens (Baart and Martens, 2012) specifications. To model the astrocyte metabolic phenotype, statistical analysis of transcriptomic data obtained from human fetal cortical astrocytes (GEO ID: GSE53404) (Malik et al., 2014) was performed. Additionally, to establish a list of genes with their respective enzymatic products, an analysis of gene expression arrays was performed using Bioconductor (Huber et al., 2015). Finally, the HMA (human metabolic atlas) (Pornputtapong et al., 2015) was used to obtain the chemical reactions associated to identified enzymes.

### Stage 2: manual refining and metabolic model reconstruction

Curation and refining of the network content was carried out in order to guarantee the presence of all enzymes of a mature human astrocyte and to ensure that the global network was balanced in mass-energy terms. In this aspect, inconsistencies in stoichiometry, reversibility and cellular location of every reaction were corrected by checking different data bases and literature (Binder et al., 2014; Kanehisa et al., 2014; Uhlen et al., 2015). Therefore, the corresponding metabolic pathways were assigned to the 3,864 reactions identified in stage one, these reactions were manually assembled to ensure network connectivity. Transport and exchange reactions were added to connect all compartments. In this sense, a schematic script (Supplementary Data 1) with this purpose was performed, so it would be possible to extract the necessary transport and exchange reactions from the HMA.

In order to assess the predictability of the generated model, a second script (Supplementary Data 2) that allowed gap detection in the entire metabolic network, identifying dead end metabolites present all over the model, was written. A dead-end is a metabolite that cannot be either produced or consumed by the reactions that are part of the network. These generated fragments of the network that are isolated due to the lack of reactions that connect them. Every “dead end” was manually examined to identify the possible reaction that would resolve the connection breach. To determine the metabolic context in which the dead end was developed, an intensive literature search using different databases such as KEGG (Kyoto Encyclopedia of Genes and Genomes) (Kanehisa et al., 2014), IntEnz (Integrated relational Enzyme database) (Fleischmann et al., 2004) and HMA (Pornputtapong et al., 2015) was carried out. Finally, gaps were solved by adding reactions that either produce or consume the dead-end metabolite. Reactions added to the model were verified in HPD (Human Protein Database) (Uhlen et al., 2015) to ensure presence of the enzyme in the astrocyte.

### Stage 3: reconstruction conversion to a predictive mathematic model based on restrictions

The metabolic reconstruction obtained in previous stages was converted into a mathematic model (stoichiometric matrix) using MATLAB 2012b^1^ (Lanz et al., 2013). To define the flux space in steady-state, restrictions were imposed based on physical-chemical principles like stoichiometric coefficient, directionality, and input/output restrictions of the different metabolites that compose the network.

All network reactions of the model were mathematically described and assembled in a stoichiometric matrix (S), where the mass balance equation system for all metabolites is represented as follows:

$$\begin{array}{l} \text{s}*\text{v}=0\\ \text{s}=m\;*\;n \end{array}$$

Where S is a stoichiometric matrix of *m* size times *n*. *m* represents the number of metabolites (3,892) and *n* the number of reactions including exchange reactions (5,659); *v* is the reaction flux vector to be identified. Restrictions for every reaction have the form:

$$\begin{array}{l} Vi\left(Lower\;limit\right)\;\leq \;V\;\leq \text{Va}\left(\text{Upper limit}\right) \end{array}$$

Where *Vi* and *Va* are represented by inferior and superior limits, respectively, which define maximum and minimum allowable flux limits for every reaction, limited between -1,000 and 1,000 (Rajkumar et al., 2016). To reduce even more the solution space, and to improve the representation of the biological phenomena under study, additional restrictions were established in the model based on astrocyte metabolic flux rates in excitatory neurotransmission (Lanz et al., 2013). Glutamate release from neuron to extracellular space and its re-uptake by astrocytes for its subsequent processing was established as the normal physiological condition for the purposes of this paper. Oxygen and glucose are the main substrates that fuel brain activity. Since these substrates normally enter extracellular space depending on the cellular metabolic state, availability of the two metabolites was considered to define the astrocyte excitatory physical condition (Shen et al., 1999).

Previous reports claim that brain glucose intake rate in a state of glutamatergic excitatory activity is 0.980 μmol/g of tissue/min (Shen et al., 1999). Specifically, glucose uptake rate by astrocytes corresponds to half the glucose that enters the brain (0.490 μmol/g of tissue/min) (Mason et al., 1995; Gruetter et al., 2001). As for oxygen, it was reported that 30% of the total is consumed by astrocytes (0.530 μmol/g of tissue/min) in the brain cortex (Zwingmann et al., 2000). Additionally, it was integrated the metabolic flux rates of the exchange reactions that are available in the literature such as lactate, carbon dioxide, ammonia, amino acids, etc. The imposed restrictions on the network are shown in Table 1.

**Table 1:** **Uptake and release rates of different metabolites in astrocyte network**.

**Constraints for the normal physiological condition**		
**Metabolite**	**Metabolic rate μmole/g/min**	**References**
Glucose	0.16	Shen et al., 1999; Gruetter et al., 2001
Linoleate	0.0011	Aureli et al., 1997
Linolenate	0.0009	Aureli et al., 1997
Histidine	0.0025	Amaral et al., 2011
Isoleucine	0.0004	Murín et al., 2009
Leucine	0.0145	Hannuniemi and Oja, 1981
Lysine	0.011	Hannuniemi and Oja, 1981
Methionine	0.0017	Lanz et al., 2013
Threonine	0.0008	Shen et al., 1999
Valine	0.0018	Westergaard et al., 1993
O2	0.515-0.530	Zwingmann and Leibfritz, 2003
Co2^**^	0.530	Zwingmann and Leibfritz, 2003
Asparagine	0.0037	Lebon et al., 2002
Tyrosine	0.0017	Lebon et al., 2002
Arginine	0.0020	Cruz and Cerdán, 1999
Glycine	0.0086	Zwingmann et al., 2000
Proline	0.0066	Zwingmann and Leibfritz, 2003
Serine	0.0016	Verleysdonk et al., 1999
Glutamate	0.232	Zwingmann et al., 2000
Ornithine	0.0031	Verleysdonk et al., 1999
Acetoacetate	0.0015	Chateil et al., 2001
Pyruvate	0.007	Verleysdonk et al., 1999
Cystine	0.0045	Shanker et al., 2001

All the constrains shown are in units of umol/min/g of proteins Asterisk (^*^) indicate release rate. These constraints were applied for all conditions in this study.

Ischemia induces energy withdrawal in astrocytes, since this injury reduces the flux of oxidative substrates such as glucose and oxygen (Thorén, 2006; Liu et al., 2015). This, in turn, slows down or stops the synthesis of ATP through glycolysis and oxidative phosphorylation (Yu et al., 2002; Rossi et al., 2007; Takano et al., 2009). To simulate ischemic conditions in the model, a reduction in the oxygen flux was performed, as well as in glucose supply. Hypoxic condition was simulated by progressive reductions of 20% over the maximum rate of oxygen consumption defined in normal physiological conditions (in a range of 0–0.530 μmol/g of tissue/min). Glucose deprivation was generated by progressive reductions of 20% over the maximum glucose intake defined in normal physiological conditions (in a range of 0–0.490 μmol/g of tissue/min). Additionally, experimental data of the flux was taken from various metabolic pathways in astrocyte cultures exposed to oxygen and glucose deprivation (Cruz and Cerdán, 1999; Amaral et al., 2011). Such procedure allowed a characterization of a feasible flux distribution for the ischemic metabolic state in astrocytes.

### Stage 4: network evaluation

Flux balance analysis (FBA) was used to adjust and evaluate restrictions imposed on the model under normal physiological conditions (Orth et al., 2010). In this aspect, the most probable metabolic reactions used by astrocyte under physiological conditions were established. Moreover, this analysis allowed to define the distributions of optimal flux and evaluate the metabolic capabilities of the astrocyte model, which ensured that the model presented in this research correctly simulated the physiological activity of an astrocyte. Therefore, the identification of metabolic capabilities of the network was not only useful to relate it with expected phenotype, but also to find the dead-end reactions when under any metabolic reaction a specific metabolite is not produced nor used by a reaction or set of reactions in the network.

To establish the metabolic functions performed by the reconstructed system, reactions known as objective functions (OF) were defined. These reactions allowed to establish the astrocytic metabolism under an physiological excitatory stimulus. Two objective functions were considered because according to literature, astrocyte metabolism its highly dependent on (1) ATP derived from energetic pathways and (2) the glutamate and glutamine exchange between astrocytes and extracellular space (Parpura et al., 2016). This objective functions are described in Table 2.

**Table 2:** **Metabolic objective functions used in the analysis of the astrocytic network**.

**Metabolic function**	**Equation**
(A) ATP production	ADP[m] + 4 H+[c] + Pi[m] = > ATP[m] + 3 H+[m] + H2O[m]
(B) Maximization of glutamate and glutamine	Glutamate[x] + Glutamine[c] = > Glutamate[c] + Glutamine[x]

*(A) Cells tend to maximize ATP synthesis or optimize the metabolic uptake of the surroundings to supply cellular demands. (B) Glutamate input and glutamine output were chosen considering the main role that the astrocyte has in glutamate detoxification of the extracellular means for its later conversion to glutamine*.

Additionally, random sampling of flux space was used to define and limit the solution space of the model in ischemic state (Wiback et al., 2004). Restrictions imposed to the solution space were given by stoichiometry, reversibility and the experimental flux measuring mentioned above. The “Random Sampling” (Wiback et al., 2004) is an algorithm implemented in RAVEN (Reconstruction, Analysis and Visualization of Metabolic Networks) (Agren et al., 2013). This algorithm encloses the region of solutions allowed in a parallelepiped with the same dimensions of the solution space (stoichiometric matrix). In this way, it generates random points on the corners of the parallelepiped that are located in a zone of possible solution. Values of the flux measures were different in the simulations of these two conditions. To calculate the difference between the fluxes and to quantify the importance of the change of every flux, an approximation based on Z score was used. To obtain Z score, standard and media deviations of every flux in each condition were obtained. Z-score is equal to the difference of the media of every one of the conditions divided into the standard deviation of this difference:

(1) $$\begin{array}{l} ZiFlux=\frac{E2\left(Vi\right)-E1\left(Vi\right)}{\sqrt{Var2\left(Vi\right)+Var1\left(Vi\right)}} \end{array}$$

The difference between the averages in the numerator follows a normal distribution (according to the theorem of the central limit) with a standard deviation equal to the standard deviation of the flux (denominator) divided by the square root of the number of samples. Therefore, Z itself follows a normal distribution with a standard deviation equal to the inverted square root of the number of samples. To keep the error lower than 0.15, five thousand simulations were performed, which reduced error probability to 0.999%. Results obtained at different biological states were validated by comparing metabolic changes with literature reports making an extended bibliographic search in databases such as OMIN (Online Mendelian Inheritance in Man) (Hamosh et al., 2005), HMDB (The Human Metabolome Database) (Wishart et al., 2013) and literature in NCBI (The National Center for Biotechnology Information)^2^.

## Results

### Characteristics of genome-scale reconstruction of the human astrocyte

**Figure 2:**
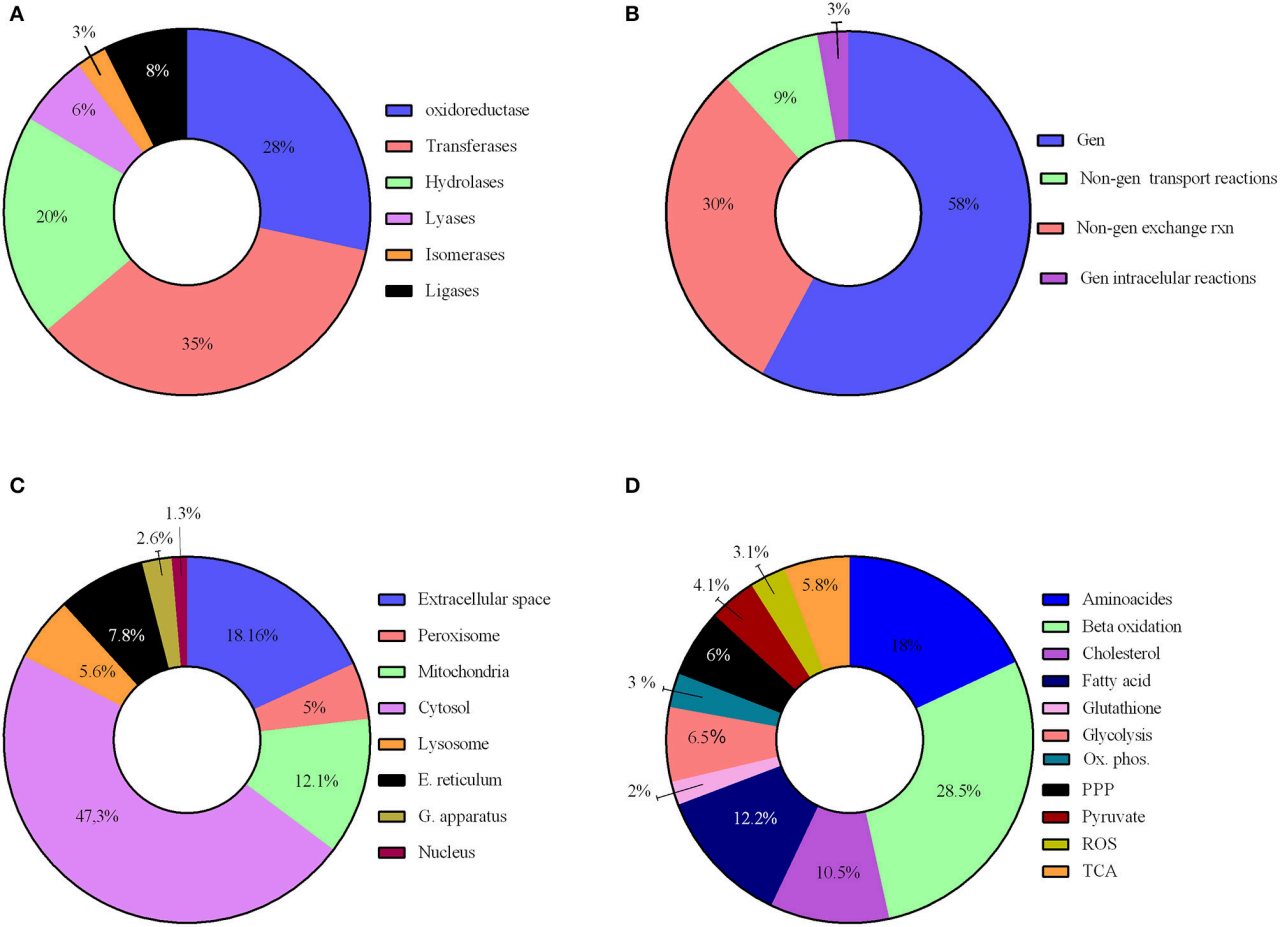
**Characteristics of the reconstructed genome scale metabolic network**. The model reconstruction has been classified on the basis of **(A)** enzymatic classification enzymatic classification (EC-number); **(B)** gene association; **(C)** subcellular locations and **(D)** Main metabolic pathways.

We have reconstructed a novel astrocyte metabolic model based on genomic, transcriptomic, biochemical and physiological data. The astrocyte metabolic network is composed by 3,765 genes, 862 enzymes, 5,007 metabolites and 5,659 reactions from which 237 are exchange reactions and 1,948 are transport reactions (Table 3). The model is available both in excel format and in SBML format as supplement (Supplementary Data 3) and uploaded in BioModels (MODEL1608180000) (Juty et al., 2015). The present model for astrocyte metabolism can be classified based on the following categories: (A) enzymatic classification (EC-number); (B) gene association; (C) subcellular locations and (D) metabolic pathways. Concerning to protein classification (EC-number), it was observed that 35% of total reactions of the model, are catalyzed by transferase enzymes such as glutathione S-transferase, glutathione peroxidase, and catalase. This group of enzymes have an essential role for astrocyte metabolic function, detoxification functions and neuroprotective capacity of astrocytes (Hertz et al., 2007). The next group of enzymes with high intervention in the model was oxidoreductases (28%), followed by hydrolases (20%), ligases (8%), lyases (6%) and isomerases (3%) (Figure 2A).

**Table 3:** **Summary of the composition of the astrocyte metabolic network characteristic**.

	**No. genes**	**No. reactions**	**No. metabolites**
Extracellular space	315	1,190	461
Peroxisome	187	381	353
Mitochondria	668	923	677
Cytosol	2,756	1,604	2,251
Lysosome	102	429	378
*E. reticulum*	369	591	530
*A. Golgi*	191	182	275
Nucleus	104	118	67
Boundary	0	0	18

Reactions were also classified on a gene-association basis. In this aspect, 58% of model reactions, have a direct association with different genes, and our results show that many are related to a wide array of brain diseases. 42% of reactions are not associated to any gene and were classified as non-associated to gene interchange reactions (30%), non-associated to gene transport reactions (9%) and non-associated to gene intracellular reactions (3%); (Figure 2B).

Regarding subcellular location, cytosolic and mitochondrial reaction contributed with 59% of the total reactions in the model. 23% of reactions belonged to peroxisome, lysosome, endoplasmic reticulum, golgi apparatus and nucleus. Transport reactions among the different organelles and extracellular space represented 18% of total reactions (Figure 2C).

Reactions were gathered around different metabolic routes (Figure 2D), being 39% of reactions included in the model part of glycolysis, pentose phosphate pathway (PPP), tricarboxylic acid cycle (TCA) and oxidative phosphorylation, which are the main energetic pathways used by astrocyte (Bouzier-Sore and Pellerin, 2013). Specific astrocyte anaplerotic reactions (Bélanger et al., 2011), such as malic enzyme and carboxylase pyruvate, were included in these pathways. 34% of the reactions were involved in metabolic pathways of biosynthesis and catabolism of different amino acids (glycine, glutamate, serine, threonine, cysteine, methionine, alanine, aspartate, and arginine). Finally, 22% of the reactions belonged to fatty acid metabolism inclusive both biosynthesis and beta oxidation. Other metabolic pathways accounted to 8% of the total reactions, including detoxification of reactive oxygen species (ROS).

### Model capabilities evaluation

**Figure 3:**
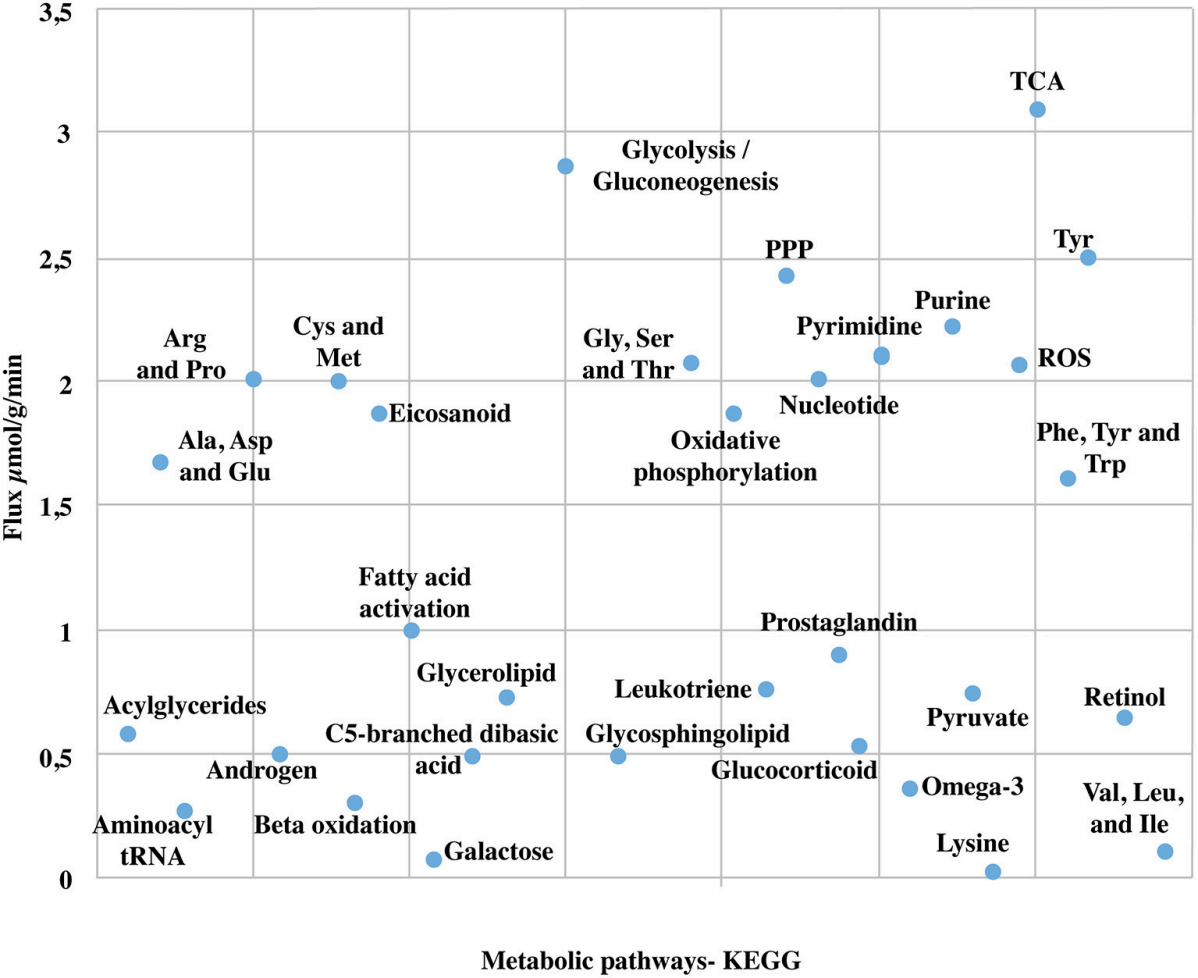
**Major metabolic fluxes (μmol/g tissue/min) in astrocyte under normal physiological conditions**. The steady-state flux space distributions of the reconstructed network were calculated through two objective functions that tested the metabolic pathways response: (1) ATP production maximization, and (2) Glutamate absorption and glutamine secretion. The abbreviations used for metabolic pathways are: TCA, tricarboxylic acid cycle; PPP, pentose phosphate pathway; Tyr, tyrosine; Gly, clycine; Ser, serine; Thr, threonine; Cys, cysteine; Met, methionine; Arg, Arginine; Pro, proline; Ala, alanine; Asp, asparagine; Glu, glutamate; Val, valine; Leu, leucine; Ile, isoleucine.

Due to the high coverage of gene-associated reactions, is possible to use this model for the integrated computational analysis of astrocyte metabolism and genomic data (Sertbaş et al., 2014). The metabolic capabilities of the reconstructed network were calculated through two objective functions. This functions, tested network response and captured metabolic scenarios developed in astrocytes in different conditions such as maximization of ATP production, glutamate absorption and glutamine release (Shen et al., 1999). Both objective functions have important physiological implications. The first one ensures the use of diverse metabolites to supply cellular demand of energy (Hertz et al., 2007). The second one ensures glutamate input and glutamine output considering that astrocyte has a main role in glutamate detoxification of the extracellular space for its later conversion to glutamine (Table 2). The results presented here were generated from flux distributions adjusted to the metabolic behavior of human astrocytes in response to maximum requirements of ATP, glutamate capture and later conversion to glutamine. The model was reconstructed in such a way that pathways of major importance to astrocyte metabolism are included (Figure 3).

### Energetic metabolism

Astrocytes play a major role in the development of energetic metabolism, given that these cells are well equipped to carry out the metabolic regulation of glucose in response to neuronal activity (Perea et al., 2014). Blood glucose captured by astrocytes can be stored as glycogen, metabolized into lactate, or finally coupled to the TCA cycle or pentose phosphate pathway (Kreft et al., 2012). When performing simulation and distributing flux space to generate a maximum ATP capacity, we observed a higher flux in oxygen and glucose uptake. Thus, glucose was mainly metabolized through glycolysis coupled to the TCA cycle and oxidative phosphorylation and consequently, activating the metabolic fluxes associated with these pathways.

We observed that the amount of glucose metabolized in the pentose phosphate pathway was highly decreased when compared to glycolysis. In this regard, the flux rate through the reaction mediated by hexokinase enzyme (EC 2.7.1.1), main modulator of phosphorylation reaction of glucose to glucose-6-phosphate, was calculated in 0.49 μmol/g/min. This value is similar to previous experimental results obtained by nuclear magnetic resonance spectroscopic approach, which reported 0.41 ± 0.03 miccromol. g(−1). min(−1) (Gruetter et al., 2001; Amaral et al., 2011). The activation of this reaction ensured fluxes through glycolysis and allowed to use the product of this reaction (glucose-6-phosphate) as substrate in several processes. Moreover, we observed flux Activation by Pyruvate kinase (EC:2.7.1.40) and lactate dehydrogenase (1.1.1.27) in physiological conditions. Pyruvate kinase catalyzes the transfer of a phosphate group from phosphoenolpyruvate (PEP) to adenosine diphosphate (ADP), yielding one molecule of pyruvate and one molecule of ATP (Ivanov et al., 2014). Consequently, ATP production is activated, as well as nicotinamide adenine dinucleotide (NADH), which is essential to keep a continuous glycolytic flux. The flux through lactate dehydrogenase (1.1.1.27), an enzyme that catalyzes pyruvate and NADH conversion to lactate and NAD+, was estimated in 0.99 μmol/g/min (Amaral et al., 2011). Likewise, flux value in the output reaction of lactate to extracellular space was 0.98 μmol/g/min (Zwingmann et al., 2000; Amaral et al., 2011). The comparison of flux proportions of glucose input and pyruvate final production in the model corresponds to what is reported experimentally by spectroscopy of C13 nuclear magnetic resonance methodologies (Schousboe et al., 1997). Importantly, under aerobic conditions, pyruvate is formed and can be metabolized via tricarboxylic acids cycle (Zwingmann et al., 2000; Kreft et al., 2012).

Regarding the activity of the tricarboxylic acid cycle (TCA), it was observed an initial metabolic flux activation by citrate synthase (EC:2.3.3.1). This enzyme catalyzes the first reaction of the acetate condensation coming from acetyl-CoA and oxaloacetate. During the aldol condensation reaction between acetyl-CoA and oxaloacetate, a thioester group (CoA) is hydrolyzed, forming citrate (Gruetter, 2002). The flux through succinate to fumarate oxidation reaction mediated by succinate dehydrogenase enzyme (EC:1.3.5.1) was calculated in 0.06 μmol/g/min, which is consistent with previous studies that estimate intracellular fluxes in primary cultures of astrocytes (Amaral et al., 2011).

Furthermore, we detected activation fluxes in oxidative phosphorylation reactions of the model. These reactions allowed to oxidize the NADH resulting from TCA to generate ATP (Mason et al., 1995). Additionally, it is possible that oxidative phosphorylation produces a small proportion of reactive species from oxygen (Murphy, 2009). Our model showed that oxidative phosphorylation produced fluxes in the reactions that were involved in the production of reactive species from oxygen, such as hydrogen superoxide and peroxide. Thus, there is a small flux activation in the ROS detoxifying reactions (Ivanov et al., 2014). The flux balance analysis (FBA) suggested an activation of pentose phosphate pathway with a production of NADPH (Supplementary Data 4). This metabolite is used as an electron donor in reductive reactions during biosynthesis or in the detoxification of hydrogen peroxides and glutathione maintenance (Bolaños et al., 2008).

### Glutamate and glutamine cycle

One of the best characterized astrocytic function is the rapid elimination of neurotransmitters released in the synaptic cleft, an essential process for the termination of transmission and maintenance of synaptic neuronal excitability (Malarkey and Parpura, 2008). Glutamate is the main excitatory neurotransmitter in the brain and it is especially critical due to its neurotoxic properties. Under normal physiological conditions, glutamate captured by cells can be used for metabolic purposes, such as protein synthesis, energetic metabolism, ammonia fixation, or as a neurotransmitter. In this sense, neurotransmitter effects of glutamate in the synapsis are highly dependent on the astrocyte (Hertz et al., 1999; Hertz and Zielke, 2004; Struzyńska, 2009). For this reasons, we evaluated the glutamate-glutamine cycle in our model, through the use of the second objective function and measurement of flux rates of glutamate uptake through the system, changing glutamate uptake from 0.03 to 0.315 μmol/g/min.

**Figure 4:**
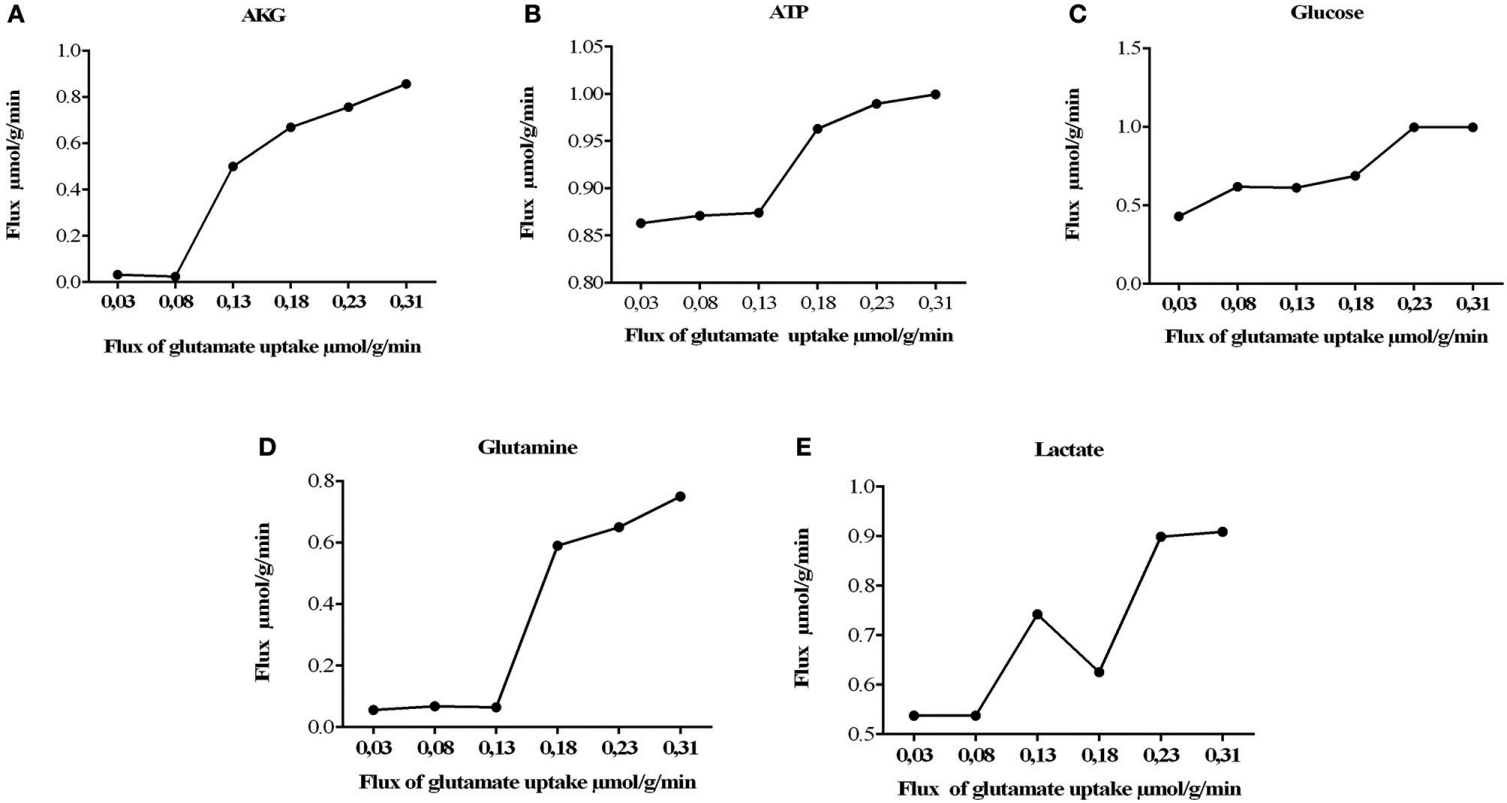
**Metabolic network states under effect of glutamate uptake under normal physiological conditions**. Histograms of the steady-state flux space are shown for glutamine, lactate, glucose, AKG (alfa-ketoglutarate), and ATP (adenosine triphosphate). During model simulation was observed that the glucose uptake, lactate, glutamine and AKG production increased with increase in glutamate (the glutamate uptake from 0, 03 to 0, 310). **(A)** Flux of alpha ketoglutarate increase dramatically, this is due to the activation of the deamination of the glutamate mediated by glutamate dehydrogenase. **(B)** activation of tricarboxilic cycle is induced by the previous obtention of AKG, as consequence ATP synthesis increases. **(C)** consumption of glucose is increased to satisfy the glycolitic state in astrocyte, however other sources of energy supply **(D)** related with the glutamine/glutamate cycle, glutamine are released to the extracellular space in order to satisfy neuronal needs. **(E)** lactate released during glutamate uptake.

**Figure 5:**
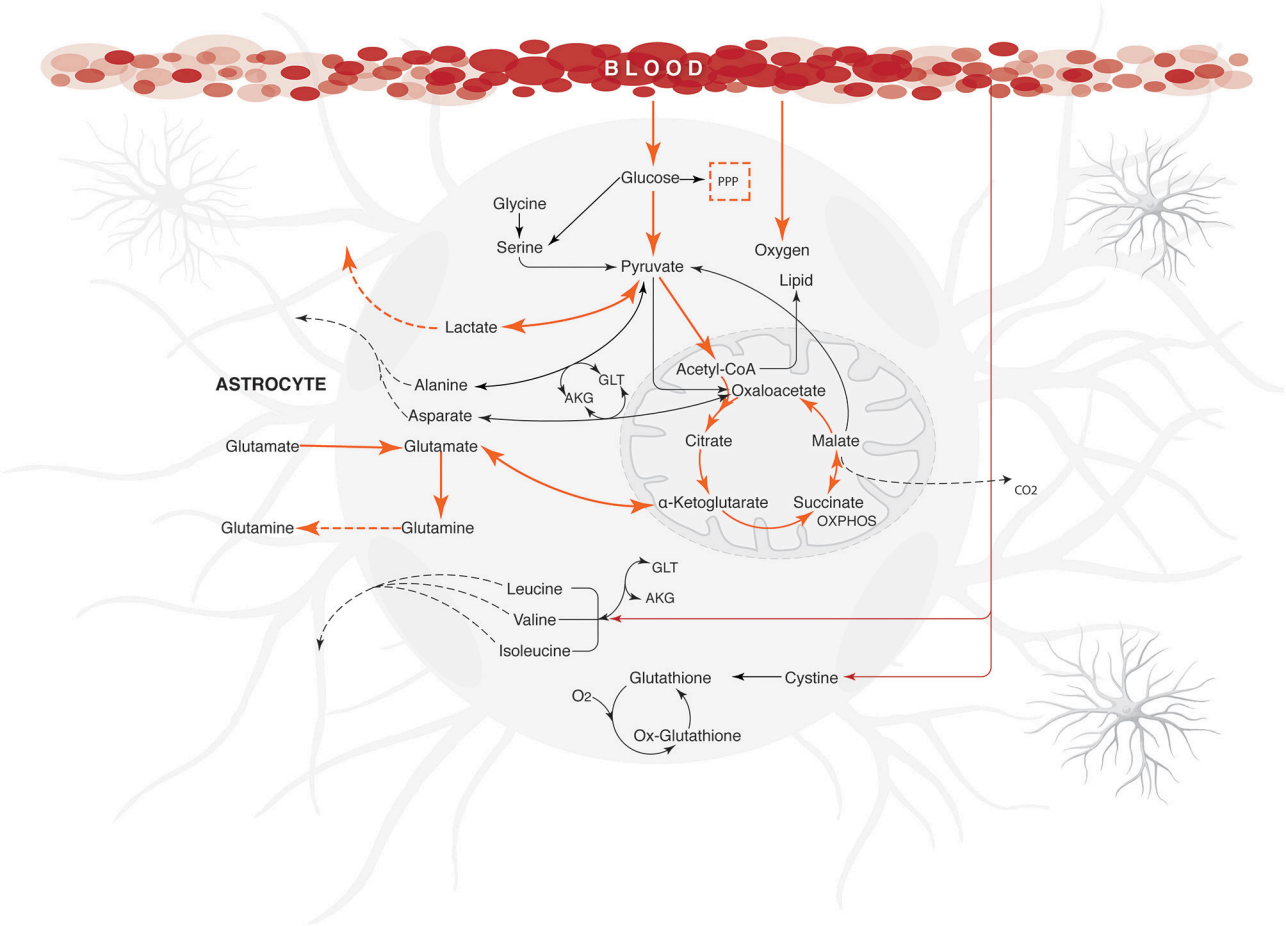
**Energetic state under normal physiological conditions in human astrocyte**. Orange arrows show the active metabolic pathways in the normal physiological astrocyte. The fluxes were calculated for maximizing the glutamate uptake /glutamine release/ ATP synthesis, using the uptake rates given in Table 1 as constraints. Dashed arrows indicate the exchange reactions for metabolic transport across the network boundary. Only key pathway fluxes are represented here for simplicity. All reactions are listed in Additional Files. The abbreviations used for metabolic pathways are: PPP, pentose phosphate pathway and Tyr, tyrosine; AKG, α-ketoglutarate; GLT, glutamate.

It was also observed that changes in glutamate uptake had a greater impact on the calculated fluxes of aerobic and anaerobic glycolysis. The fluxes in glucose production and release of lactate increased as well, showing that our model is in agreement with studies carried out on primary cultures of cerebral cortical astrocytes, which measured these metabolites by enzymatic and autoradiography techniques (Pellerin and Magistretti, 1994). Likewise, glutamate uptake reduction was correlated with a decrease in the metabolic flux exerted by glutamine synthetase (EC 6.3.1.2), glutamate dehydrogenase (EC 1.4.1.) and lactate dehydrogenase (1.1.1.27) (Figure 4). Finally, the AKG production was proportionality increased with glutamate uptake (from 0.0003 to 0.335 μmol/g/min). These results are in agreement with previous glutamate labeling experiments, in astrocytic primary cultures (Mason et al., 1992; Schousboe et al., 1997; Chatziioannou et al., 2003). Figure 5 shows the main active pathways during simulation in normal physiological conditions.

### Potential of reconstructed model in the analysis of pathological states

Brain ischemia results from stroke or head trauma, causing a decrease in the brain energy supply that in some instances can produce irreversible damage, being one of the main causes of death worldwide (Hertz et al., 1999; Lebon et al., 2002). As mentioned before, during ischemic strokes, astrocytes play an important role in the protection of affected tissues given their improved resistance to pathophysiological conditions and thus helping in the protection of spared tissue from further damage, by the production of growth factors, antioxidants and glutamate uptake (Swanson et al., 1997). Currently, the precise metabolic determination of astrocytes during pathologies is chalenging using experimental approaches. Thus, computational modeling under restrictions can help to understand the global metabolic changes and cellular functions that take place in different pathologies. Given the importance of ischemia in astrocytes, an ischemic episode was simulated and evaluated in our model. To simulate ischemic conditions, a reduction in oxygen and glucose flux was performed by progressive reductions of 20% over the maximum rate of oxygen consumption defined in normal physiological conditions (in a range of 0–0.530 μmol/g of tissue/min) (Mason et al., 1995; Shen et al., 1999). Likewise, glucose deprivation was generated by progressive reductions of 20% over the maximum glucose intake defined in normal physiological conditions (in a range of 0–0.490 μmol/g of tissue/min). Flux distributions throughout the network were calculated using Monte Carlo Sampling (Random Sampling) (Wiback et al., 2004; Bordel et al., 2010). We took literature data of different metabolic fluxes in astrocyte cultures exposed to oxygen and glucose deprivation (Cruz and Cerdán, 1999; Amaral et al., 2011). Such procedure allowed limiting flux space of simulation, therefore flux values generated were more accurate and allowed characterization of a feasible flux distribution for the ischemic metabolic state in astrocytes.

### Ischemic conditions

**Figure 6:**
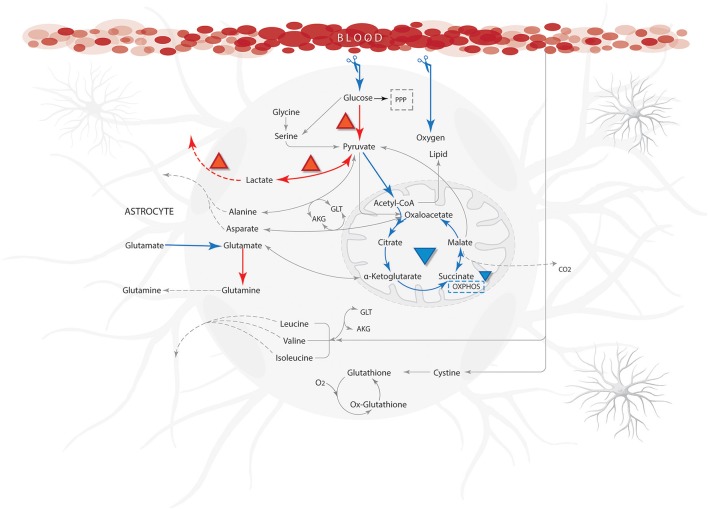
**Qualitative properties for ischemic from literature compared with model results**. Blue arrows show reactions with low flow or not activated and the orange arrows indicate a high flow during simulation. The abbreviations used for metabolic pathways are: PPP, pentose phosphate pathway and Tyr, tyrosine; AKG, α-ketoglutarate; GLT, glutamate.

Flux distributions on ischemic conditions were compared with distributions calculated on the normal physiological state (Supplementary Data 4). The results showed a significant flux reduction of the reactions grouped in the metabolic pathways of fatty acids oxidation, tricarboxylic acid cycle, oxidative phosphorylation and glutamate-glutamine cycle. Likewise, increases in fluxes through anaerobic glycolysis and pentose phosphate pathways were observed (Figure 6). Previous studies have shown that substrate limitations cause acceleration of flux through these pathways (anaerobic glycolysis and pentose phosphate pathway) and depression in the others (fatty acids oxidation, tricarboxylic acid cycle, oxidative phosphorylation and glutamate-glutamine cycle) (Swanson et al., 1997).

Mitochondria are the main organelles of ATP generation at cellular level, and are mostly dependent on oxygen and glucose reservoirs (Baker et al., 1998). Decreases in oxygen fluxes, highly reduced the ATP production in the (Weisbrot-Lefkowitz et al., 1998). Moreover, when evaluating different reactions performed in the mitochondria, an important decrease in the reaction fluxes within the TCA and oxidative phosphorylation was observed. Importantly, the decrease in flux distributions in these processes are general characteristics of astrocytes under ischemic conditions (Sonnewald et al., 1994; Swanson et al., 1997; Niitsu et al., 1999). Moreover, studies in primary cultures have also shown that the dysfunction of Na+K+ATPase pump due to ATP depletion is a fundamental event in brain ischemia (Yu et al., 2002). Consequently, the inhibition of this pump during ischemia causes a loss of cellular ionic gradients and membrane depolarization (Chateil et al., 2001). In this aspect, our simulation showed that Na+/K+ATPase flux decreased proportionally with ATP reduction.

**Figure 7:**
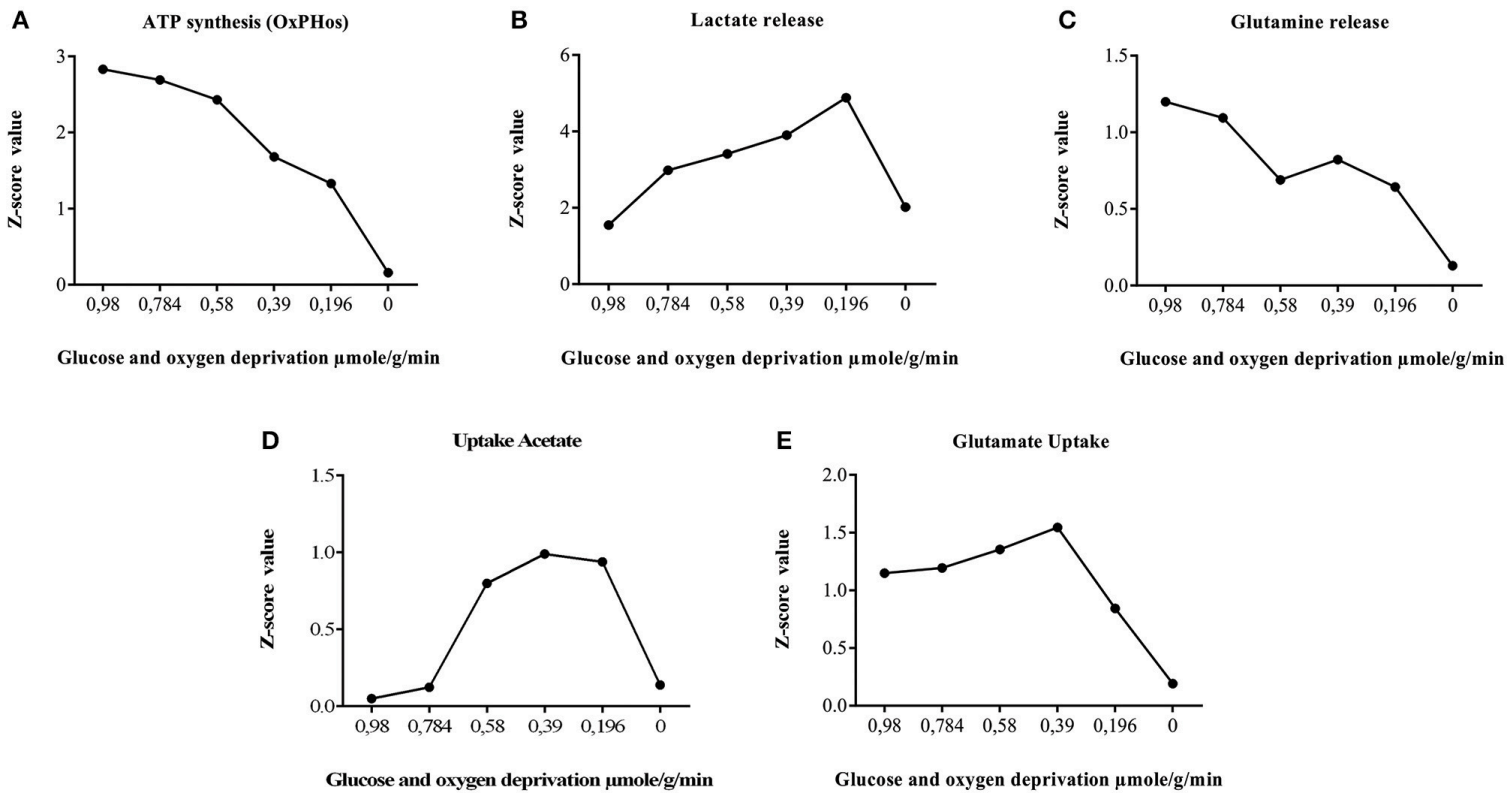
**Astrocyte ischemia**. Effect of glucose and oxygen deprivation of astrocytes on metabolic fluxes rates calculated with random sampling approach. The global result, showed a significant increase in the tendency of the flux through anaerobic glycolysis and pentose phosphate pathway. Similarly, a reduction of the flux of the reactions grouped in the metabolic routes of fatty acids oxidation, tricarboxylic acid cycle, oxidative phosphorylation, glutamate-glutamine cycle, and Na+/K+ATPasa. **(A)** privation of sources of oxygen and glucose decrease gradually the synthesis of energy, until the model is unable to satisfy requirements. **(B)** synthesis of lactate is useful as replacement of energy until there are no sources of energy that supply pumps to interchange other metabolites. **(C)** pumps and transporters fluxes decrease with the energy depletion, among them glutamine **(D)** Acetate as an alternative source of energy is useful for model until transporters and pumps did not work by the lack of energy. **(E)** Detoxification properties are decreased following the energetic depletion.

Regarding the behavior of anaerobic glycolysis reactions in simulations, we observed that despite the increased flux in this pathway, ATP production was notoriously diminished. This may occur because cell energy balance is compromised due to the reduction of glucose metabolism and decrease of substrates needed for ATP production (oxygen and glucose). In this aspect, respiration is inhibited but glycolysis continues in astrocytes causing an accumulation in lactate and protons and a rapid intracellular acidification (Kawamata et al., 1995). When lactate behavior is assessed in the model, we found an increased flux on lactate production reactions (Figure 7); similar to previous experimental studies (Dienel and Hertz, 2005). This metabolic behavior can occur when glycolytic anaerobic pathway in highly activated to compensate for the partial inactivation of TCA cycle that happens in mitochondria (Sonnewald et al., 1994).

On the other hand, the extracellular concentration of glutamate is increased during brain ischemia. In this sense, glutamate uptake by astrocytes is important in the regulation of ischemic state. In ischemic conditions, the reduced energy status in astrocytes can lead a disruption in the glutamate-glutamine cycle (Dallas et al., 2007). In the simulation, we observed that glucose and oxygen deprivation leads to the decrease in glutamate uptake flux and glutamine release. As expected, glutamate uptake flux falls into much lower levels during a severe ATP depletion, indicating that glutamate internalization into the cell is mediated directly by energetic requirements (Figure 7). The results of the simulation are in accordance with studies carried out in astrocytes subjected to hypoxia (Swanson, 1992; Chateil et al., 2001; Dallas et al., 2007).

Certain metabolic intermediaries, during ischemic circumstances, can replace glucose and play a role as alternative energy substrates (Chateil et al., 2001). One example is acetate, which is an alternative substrate transported only to glial cells. Acetate is its converted to acetyl-CoA by acetyl-CoA synthetase (acetate–CoA ligase; EC 6.2.1.1), and then enters the TCA cycle by condensing with oxaloacetate to form citrate. (Castelló-Ruiz et al., 2014). Regarding the acetate uptake, our model showed an increase in acetate absorption in response to oxygen and glucose reduction, compared with normal conditions (Figure 7). Moreover, in the model, we observed an increased flux in antioxidant enzymes and key enzymes in glycolysis (Table 4). In this aspect, many studies have shown that these enzymes can be part of a machinery for cultured astrocytes to adapt to an ischemic environment, suggesting that the overexpression of antioxidant enzymes may be an important protective mechanism that prevents brain injury (Sonnewald et al., 1994; Niitsu et al., 1999; Grelli et al., 2013). Overall, the *in silico* predictions of our model, are similar to those experimentally reported in ischemic astrocytes, suggesting the predictive value of the present model.

**Table 4:** ***In silico*** **predictions of changes in metabolic functions in ischemic conditions were compared with those reported in the literature**.

**Name enzyme**	**E.C number**	**Model prediction**	**Experimental data**	**References**
Catalase	EC:1.11.1.6	Up	Up	Gu et al., 2004; Armogida et al., 2011
Superoxide dismutase	EC:1.15.1.1	Up	Up	Baker et al., 1998
Glutathione peroxidase	EC:1.11.1.9	Up	Up	Weisbrot-Lefkowitz et al., 1998
Glyceraldehyde-3-phosphate dehydrogenase	EC:1.2.1.12	Up	Up	Tanaka et al., 2002
Hexokinase	EC:2.7.1.1	Up	Up	Niitsu et al., 1999
Nicotinamide phosphoribosyltransferase	EC:2.4.2.12	Up	Up	Yamauchi et al., 2001; Wang et al., 2011; Jing et al., 2014; Morris-Blanco et al., 2014
Phospholipase A2	EC:3.1.1.4	Up	Up	Muralikrishna Adibhatla and Hatcher, 2006
Isocitrate dehydrogenase	EC:1.1.1.42	Down	Down	Grelli et al., 2013
Monoamine oxidase	EC:1.4.3.4	Down	Down	Khvatova et al., 1997, 2007

*Overall, in silico predictions of changes in metabolic function are similar to the changes reported experimentally in practices performed in astrocytes submitted to ischemia*.

## Discussion

A comprehensive model of astrocyte metabolism was generated based on the analysis and integration of genomic, transcriptomic, biochemical and physiological data, followed by manual curation. Compared with previous metabolic models, this reconstruction model is to our knowledge, the most complete mathematical structured database (in term of metabolites/genes and reactions), which would enable systematic studies of human astrocyte metabolism. This model includes the central routes of carbohydrate metabolism, energetic metabolism, purine metabolism, aminoacids metabolism, vitamin and cofactor metabolism, glutamate-glutamine cycle, lipid metabolism and glutathione metabolism.

We studied the flux distribution in these pathways. For this goal, two simulations were generated from this reconstruction, representing the astrocyte in normal physiological state and ischemic conditions. Through these simulations, we tried to gain some insight into the metabolic pathways and metabolites that were present in these metabolic states. In this aspect, the model was capable of yielding results, which were in correspondence to the experimentally proved phenomena.

By setting the objective function, the model was demanded to generate the maximum energy production. Therefore, increased fluxes in energetic pathways were observed. Specifically, a higher activation in the TCA cycle pathway was observed, followed by glycolysis, pentose phosphate and oxidative phosphorylation, showing direct relations among these different cycles. Our results shown glycolytic flux activation in response to an abrupt energetic demand (e.g., glutamatergic neuronal excitation) (Fu and Jhamandas, 2014; Ivanov et al., 2014). The model can respond with the metabolic machinery to produce ATP, showing oxidative activity and increasing energetic substrate consumption. These results agree with recent studies reporting that astrocytes express transcripts that predict self-sufficiency in glycolysis as well as in oxidative metabolism (Lovatt et al., 2007). Moreover, most enzymes participating in the tricarboxylic acids cycle (TCA) are expressed in high levels in astrocytes (Panov et al., 2014), which was evident in the model.

Flux distributions in simulations were related to the metabolic tasks set in the model. For example, in a excitatory physiological cellular state, the model responded by maximizing energy production in response to the requirements generated by the glutamate uptake. Experimental evidence suggest that astrocytes present a high flux rate at glycolysis level, making them “glycolytic cells” (Fu and Jhamandas, 2014; Ivanov et al., 2014); however, they also have an important oxidative metabolism especially toward glutamate (Bouzier-Sore and Pellerin, 2013). Normal physiological glutamatergic state was simulated in astrocyte by using restrictions and metabolic tasks according to the studied system (simultaneous maximization of the glutamate/glutamine cycle and ATP production). In this simulation, we observed the relationship between glutamate uptake and central metabolism, which is consistent with the metabolic mechanisms reported in the literature. Experimental evidence suggest that once a neuron has released glutamate to the synaptic cleft, the astrocytes uptake the glutamate using several transporters, e.g., excitatory amino acids transporters (EAAT); for this transport, a symport between glutamate and sodium is required, then a Na^+^, K^+^-ATPase supplies this ion inside the astrocyte (Allaman et al., 2011). However, the latter requires a flux of ATP that is supplied by the glycolytic and oxidative pathway of the astrocyte (Schousboe et al., 2014).

The model also showed a high flux in glutamine synthetase compared with other glutamate metabolizing enzymes, suggesting that glutamate is predominantly converted into glutamine due to an ATP-dependent reaction. Furthermore, the increase in ATP production and glucose was related with the increase in glutamate uptake (Figure 4). These observations are consistent with the research of Pellerin and Magistretti, who showed that glutamate stimulates glycolysis and ATP in cultures of astrocytes (Magistretti and Pellerin, 1999). Glutamate, despite being a neurotransmitter, also participates as a metabolite to become in alpha ketoglutarate (Lying-Tunell et al., 2009; Ivanov et al., 2014), it is incorporated in the TCA cycle to perform the metabolic process of recycling pyruvate (Figures 4A,E) and ATP production (Dienel, 2013).

Using radiolabeled glutamate, McKenna et al. (2009) and Sonnewald determined through the production of ^14^CO_2_, that extracellular glutamate could be metabolized to glutamine or enter the TCA cycle as alpha ketoglutarate by transamination mediated by the aspartate aminotransferase, as well as by direct oxidation mediated by glutamate dehydrogenase (Grelli et al., 2013; Castelló-Ruiz et al., 2014; Fu and Jhamandas, 2014). Regarding the pyruvate cycle, it has also been determined by radiolabeling that glutamate upon entering the TCA cycle can be oxidized to malate or oxaloacetate by malic enzyme or the combined action of phosphoenolpyruvate carboxykinase and pyruvate kinase (McKenna, 2013). On the other hand, the production of lactate and glutamine was increased as a normal mechanism of astrocyte (Figures 4D,E), for example to supply the neuron with metabolites and neurotransmitters (Allaman et al., 2011).

The primary insult to which brain cells are exposed during ischemia is the decrease on substrates such as glucose and oxygen. A critical event, considering that brain cells require a continuous supply of oxygen and energetic substrates for maintenance of its functional and structural integrity (Castelló-Ruiz et al., 2014; Kuroiwa et al., 2016). Astrocytes have multiple functions in several metabolic aspects of ischemic brain damage, because they are capable of protecting neurons against numerous insults, and in that way promoting neuronal survival. In this aspect, astrocyte dysfunction may lead to an alteration of the normal homeostasis between neuron and astrocyte, causing metabolic changes with consequent cell death.

Ischemia is an example in which astrocytes seem to play a “paradoxical role” in neuronal survival (Molofsky et al., 2012). Severe ischemia leads to an undersupply of oxygen, glucose and energy, for astrocytes. Brain damage during and after ischemic events is associated with reduced energy production (ATP), decreased glutamate clearance, and increased acidosis due to an increase in lactate production (Rossi et al., 2007). In consequence, mechanisms of ATP depletion, intracellular acidification and generation of reactive oxygen species are involved in the pathophysiology and severity of cell damage in ischemia (Rossi et al., 2007). Several experimental studies have shown that glycolitic enzymes are regulated under ischemia, in order to stabilize ATP levels and protect neuronal survival (Cruz and Cerdán, 1999; Dienel and Hertz, 2005; Rossi et al., 2007). Although the model did not allow any prediction over time course, because kinetic values were not taking into account, our results emphasized the possible metabolic change in the different foci of ischemia (Swanson et al., 1997).

Our model also showed a progression in the activation of astrocytic protective effects during ischemic state (Figure 6). While the amount of ATP production decreased, the activity of some reactions that required this substrate were limited. Then, the astrocytic metabolism changed toward metabolic alternatives that allowed cell survival or the feasible solution in the stoichiometric matrix. One of these alternatives was an increase in lactate production (Figure 7C), which allowed NAD^+^ recovery and ATP production from glycolysis. Subsequently, enzymes of the glycolytic pathway became activated, specifically, hexokinase (EC 2.7.1.1) and glyceraldehyde 3-phosphate dehydrogenase (EC:1.2.1.12). The latter not only catalyzes the sixth step of glycolysis, but also has been involved in non-metabolic processes including transcription, apoptosis and axonal transport (Tanaka et al., 2002; Suzuki et al., 2015). Thus, production of lactate increased to satisfy the energy requirements by NAD^+^, because astrocytes can produce lactate for neuronal oxidative phosphorylation in response to ischemic state in order to maintain continuous neurotransmission (Lying-Tunell et al., 2009). To meet this requirement, astrocytes support neurons by balancing different glycolytic fluxes (Ivanov et al., 2014). Consequently, compensatory glycolysis does not prevent the decrease in ATP level during the last stages of ischemic state simulation.

One of the best-characterized functions of astrocytes is the quick removal of neurotransmitters released into the synaptic cleft, an essential process for the termination of synaptic transmission and maintenance of neuronal excitability (Guillamón-Vivancos et al., 2015). The decrease of ATP in the model has severe consequences in this metabolic process, because the Na, K-ATPase requires a constant source of ATP, and for this reason the release or uptake of different metabolites can be altered. Additionally, glutamate transport requires sodium which is provided by these pumps (Hertz et al., 1999; Danbolt, 2001). During ischemia, Sodium pump failures are associated with the raise of extracellular concentration of glutamate, which triggers excitotoxicity. These results are consistent with the hypothesis that ischemia leads to lower glutamate transport in astrocytes (Sonnewald et al., 1994; Anderson and Swanson, 2000). Model simulated ischemia (i.e., zero uptake of oxygen and glucose), highly reduced the glutamate uptake and glutamine release. During the initial steps of ischemia both, Na^+^,K^+^-ATPase and glutamate transporters are overexpressed as a survival mechanism, which correlates with a rise in glutamate uptake (Figure 7E) (Kim et al., 2013).

During severe ischemia, there is an increased astrocytic dead, as seen in Figure 7, where in the total deprivation of glucose and oxygen the decay of any other process is absolute (Figures 7A–E). Additionally, we have also identified changes in some antioxidant enzymes, such as catalase, superoxide dismutase and Glutathione peroxidase. Recent studies have shown that astrocytes maintain high antioxidative concentrations, making them more resilient to oxidative stress than neurons. For that reason, under ischemic conditions astrocytes increases the expression of antioxidant enzymes (Raps et al., 1989; Semsei et al., 1991; Espinosa-Diez et al., 2015; Finsterwald et al., 2015). Additionally, other enzymes related with mitochondrial dysfunction such as Isocitrate dehydrogenase (EC:1.1.1.42) and monoaminoxidase (EC:1.4.3.4), had been related with a decrease in their activity, first is associated to the lack of NAPD^+^ inside the cell, and the latter with lower levels of oxygen inside the cell which inhibits oxidative deamination (Khvatova et al., 1997, 2007).

In conclusion, we generated the most complete metabolic reconstruction of an astrocytic to the date. In this aspect, the simulation of different conditions in or astrocytic model were positively correlated with previous studies (Danbolt, 2001; Allaman et al., 2011; Jackson et al., 2014); showing that the *in silico* astrocyte model presented here, is able to represent metabolic behaviors and simulates changes in metabolic fluxes in response to normal physiological state and ischemia. This reconstruction is fully compatible with the Human metabolic atlas, enabling the of astrocyte-cells interactions. In this sense, this metabolic model will also allow the scientific community to dynamize the identification of metabolic routes associated to different phenotypes (aging diseases, injuries, etc.), identification of active therapeutic targets, discovering of key proteins in response to an insult (metabolic, deprivation, etc.), future drug evaluations and determination of biomarkers in different neurologic diseases such as Alzheimer, Parkinson, Huntington, Amyotrophic Lateral Sclerosis, Multiple Sclerosis, Schizophrenia, among others. The study of the metabolic behavior of these diseases during aging will help to understand characteristics of receptors, transporters and pumps that are vital in the development of these processes (De Lores Arnaiz and Ordieres, 2014). Finally, we demonstrated the high-quality astrocyte model is very well suited for integration of omics data and hereby result in a compressive understanding of astrocyte biology in response different metabolic phenotypes.

## Author contributions

CM and JG designed the methods and simulations; CM and DS performed the simulations; CM, DS, GB, and JG analyzed the data; CM, JG, and DS wrote the manuscript.

### Conflict of interest statement

The authors declare that the research was conducted in the absence of any commercial or financial relationships that could be construed as a potential conflict of interest.

## Footnotes

^1^The MathWorks, Inc. MATLAB and Statistics Toolbox Release 2012b.

^2^http://www.ncbi.nlm.nih.gov/.
