# Supplementary material for: Genome-Scale Reconstruction of the Human Astrocyte Metabolic Network
Source: Front Aging Neurosci. 2017 Feb 13;9:23. doi: 10.3389/fnagi.2017.00023 (PMC5303712; doi:10.3389/fnagi.2017.00023)
Supplement: Supplementary file 1 [file DataSheet1.docx]

Supplementary data 1

#!/bin/bash

while read line

do

grep -w "$line" reacciones_transporte.data

done < $1 >> transporte.txt

sort transporte.txt | uniq > transporte_uniq.data

rm transporte.txt
