# Supplementary material for: Genome-Scale Reconstruction of the Human Astrocyte Metabolic Network
Source: Front Aging Neurosci. 2017 Feb 13;9:23. doi: 10.3389/fnagi.2017.00023 (PMC5303712; doi:10.3389/fnagi.2017.00023)
Supplement: Supplementary file 2 [file DataSheet2.docx]

Supplementary data 2

#!/bin/bash

output="Resultados.data"

rm $output

awk -F= '{ if (/<=>/) {print $1, $2} else {print $2}}' $1 > productos.data

awk -F= '{ if (/<=>/) {print $1, $2} else {print $1}}' $1 > reactivos.data

cat productos.data | sed 's/> //g' | sed 's/ </\n/g' | sed 's/ + /\n/g' | sed 's/^[0 -9 ]* //g' > productos1.data

sort productos1.data | uniq -c >> $output

sort productos1.data | uniq > productosfinal.data

cat reactivos.data | sed 's/ <//g' | sed 's/> /\n/g' | sed 's/ + /\n/g' | sed 's/^[0 -9 ]* //g' | sed 's/ *$//g' > reactivos1.data

sort reactivos1.data | uniq -c >> $output

sort reactivos1.data | uniq > reactivosfinal.data

echo -e "\n********Metabolitos Desconectados********" >> $output

cat productosfinal.data reactivosfinal.data > msc.txt

cat msc.txt | sort | uniq -u >> $output
